# Phase 1 study of IMCnyeso, a T cell receptor bispecific ImmTAC targeting NY-ESO-1-expressing malignancies

**DOI:** 10.1016/j.xcrm.2025.101994

**Published:** 2025-03-06

**Authors:** Juanita S. Lopez, Mohammed Milhem, Marcus O. Butler, Fiona Thistlethwaite, Brian A. Van Tine, Sandra P. D’Angelo, Melissa L. Johnson, Takami Sato, Hendrik-Tobias Arkenau, Ramakrishna Edukulla, Jason Wustner, Shannon Marshall, Jordi Rodon

**Affiliations:** 1 Drug Development Unit, Institute of Cancer Research and the Royal Marsden Hospital, London SW7 3RP, UK; 2 Department of Internal Medicine, Division of Hematology/Oncology and BMT, University of Iowa Hospitals and Clinics, Iowa City, IA 52242, USA; 3 Department of Medical Oncology and Hematology, Princess Margaret Cancer Centre, Toronto M5G 2M9, ON, Canada; 4 Departments of Medicine and Immunology, University of Toronto, Toronto M5G 2M9, ON, Canada; 5 Division of Cancer Sciences, Faculty of Biology, Medicine and Health, The University of Manchester, Manchester M20 4GJ, UK; 6 Department of Medical Oncology, The Christie NHS Foundation Trust, Manchester M20 4BX, UK; 7 Division of Oncology, Washington University in St. Louis School of Medicine, St. Louis, MO 63110, USA; 8 Department of Medicine, Memorial Sloan Kettering Cancer Center, New York, NY 10065, USA; 9 Department of Medicine, Weill Cornell Medical College, New York, NY 10065, USA; 10 Lung Cancer Research Program, Sarah Cannon Research Institute at Tennessee Oncology, Nashville, TN 37203, USA; 11 Department of Medical Oncology, Sidney Kimmel Comprehensive Cancer Center, Jefferson University, Philadelphia, PA 19107, USA; 12 Phase-1 Trials Unit, Sarah Cannon Research Institute, UCL Cancer Institute, University College London, London W1G 6AD, UK; 13 Biometrics, Immunocore, Gaithersburg, MD 20878 USA; 14 Translational Medicine, Immunocore, Conshohocken, PA 19428 USA; 15 Clinical Development, Immunocore, Gaithersburg, MD 20878 USA; 16 Department of Investigational Cancer Therapeutics, The University of Texas MD Anderson Cancer Center, Houston 77030, TX, USA

**Keywords:** immunotherapy, solid tumors, dose escalation, ImmTAC, T cell receptor, bispecific, synovial sarcoma, cancer-testis antigen, NY-ESO

## Abstract

IMCnyeso, an immune mobilizing monoclonal T cell receptor against cancer (ImmTAC) bispecific (New York esophageal squamous cell carcinoma [NY-ESO]×CD3) T cell engager, targets an NY-ESO-1/L-antigen family member-1 isoform A (LAGE-1A) peptide presented by histocompatibility leukocyte antigen (HLA)-A∗02:01. In this phase 1 study, 28 HLA-A∗02:01+ patients with advanced NY-ESO-1/LAGE-1A-positive advanced tumors (*n* = 28) receive IMCnyeso weekly intravenously (dose range: 3–300 μg; 7 dose-escalation cohorts). The primary objective is to identify the maximum tolerated dose (MTD) or recommended phase 2 dose (RP2D); additional objectives include preliminary anti-tumor activity, pharmacokinetics, immunogenicity, and pharmacodynamic changes. The study was terminated before fully enrolling dose escalation, and the MTD was not identified. There are no treatment-related discontinuations or deaths. The most common adverse events are grade 1/2 cytokine release syndrome and associated symptoms. Cytokine induction and transient lymphocyte count decreases are observed at doses 30–300 μg. At these doses, preliminary efficacy includes mixed response (2 patients) and a median overall survival of 12 months. IMCnyeso is well tolerated and, at doses ≥30 μg, induces pharmacodynamic changes consistent with T cell redirection. This study was registered at ClinicalTrials.gov (NCT03515551).

## Highlights

• IMCnyeso is an ImmTAC bispecific T cell engager
• IMCnyeso targets NY-ESO-1/LAGE-1A-positive tumors
• IMCnyeso had a manageable safety profile with no treatment-related discontinuations
• IMCnyeso induced pharmacodynamic changes and anti-tumor activity at doses ≥30 μg

## Graphical abstract

**Figure:**
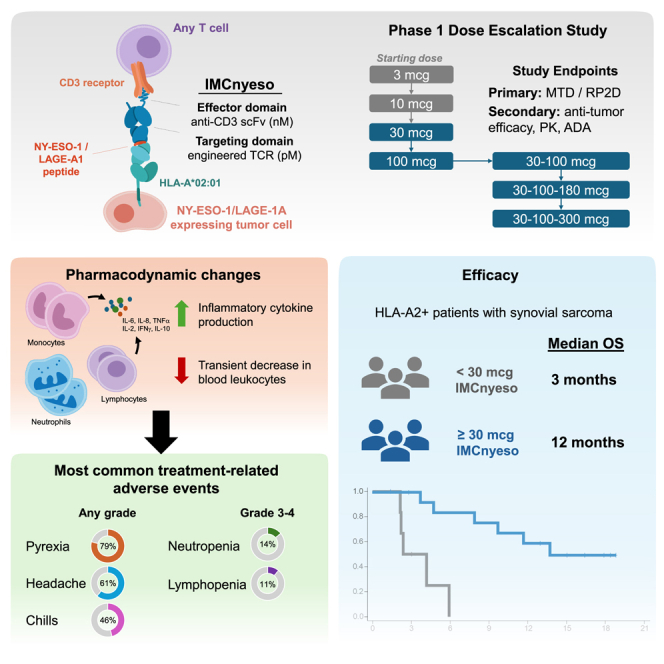

## Teaser

In this phase 1 dose-escalation study, Lopez et al. report the manageable safety profile of IMCnyeso, an ImmTAC bispecific T cell engager, in patients with NY-ESO-1/LAGE-1A-positive advanced tumors. Pharmacodynamic activity and promising overall survival in patients with synovial sarcoma were observed at doses above 30 μg.

## Introduction

New York esophageal squamous cell carcinoma 1 (NY-ESO-1) is a cancer-testis antigen that is normally expressed in germ cells and placental cells and aberrantly expressed in some malignancies. An advantage of targeting cancer-testis antigens such as NY-ESO-1 is that expression is tumor specific, minimizing normal cell toxicity. Spontaneous humoral and cellular immune responses against NY-ESO-1 have been observed in patients with cancer but not healthy donors, leading to the evaluation of NY-ESO-1-targeted immunotherapies, including vaccines and adoptive T cell therapies.^1^^,^^2^

During the course of this study, two cellular therapies have demonstrated the benefit of directing engineered T cells against cancer-testis antigens in synovial sarcoma and other advanced cancers. In a phase 1 trial, letetresgene autoleucel (lete-cel, GSK3377794), a T cell receptor (TCR)-engineered T cell (TCR-T) targeting NY-ESO-1/L-antigen family member-1 isoform A (LAGE-1A), reported an overall response rate per RECIST v.1.1 of 33% (15/45) in synovial sarcoma.^3^ A second TCR-T targeting the cancer-testis antigen melanoma-associated antigen 4 (MAGE-A4), afamitresgene autoleucel (afami-cel, ADP-A2M4), was recently approved based on phase 2 data reporting an overall response rate of 39% (19/52) in patients with advanced synovial sarcoma and myxoid round cell liposarcoma.^4^

ImmTAC (immune mobilizing monoclonal T cell receptor against cancer) molecules are a class of T cell redirecting bispecific fusion proteins that use an affinity-enhanced TCR to target any protein, including intracellular antigens, that is processed and presented as peptide-histocompatibility leukocyte antigen (HLA) complexes on the target cell surface.^5^ Once bound to its specific peptide-HLA on the surface of tumor cells, these ImmTAC molecules can redirect and activate any T cell, via CD3 engagement, to produce effector cytokines and/or kill the antigen-presenting cancer cell.^6^ In addition, ImmTAC-mediated tumor cell lysis may help to prime an endogenous anti-tumor immune response in a process known as epitope spread.^7^

This study evaluated IMCnyeso, an investigational ImmTAC comprising a soluble, affinity-enhanced, HLA-A∗02:01-restricted TCR specific for a peptide (SLLMWITQC) derived from NY-ESO-1^8^ and its homolog, LAGE-1A,^9^ fused to a nanomolar affinity anti-CD3 single-chain variable fragment (scFv) effector domain. IMCnyeso binds its target peptide-HLA with an affinity of ∼50 pM and a binding half-life of several hours.^10^ In preclinical assays, IMCnyeso was found to induce cytokine production and dose-dependent tumor cell lysis by CD8^+^ effector T cells in co-cultures with antigen-positive tumor cell lines but not in co-cultures with antigen-negative tumor cells or healthy tissue cells. IMCnyeso was also demonstrated to prevent the growth of new and established NY-ESO-1-expressing tumors in humanized mouse xenograft models.^10^

The approach of using a TCR bispecific to redirect polyclonal T cells against tumor cells has been validated by tebentafusp (gp100 × CD3), which demonstrated an overall survival (OS) benefit (hazard ratio 0.51) versus investigator’s choice of pembrolizumab, ipilimumab, or dacarbazine in patients with previously untreated metastatic uveal melanoma.^11^ Progression-free survival (PFS) was also in favor of tebentafusp (hazard ratio 0.73), but both PFS and the RECIST v.1.1 overall response rate of 9% underestimated the observed OS benefit.

## Results

### Patient population

A total of 508 patients were pre-screened, including 236 patients with melanoma (81 patients with uveal melanoma), 133 patients with non-small cell lung cancer (NSCLC), 103 patients with synovial sarcoma, and 36 patients with urothelial carcinoma. Almost half (*n* = 242, 48%) had an HLA-A∗02:01 allotype, and 49 of the 242 were positive for NY-ESO-1 and/or LAGE-1A. Pre-screening success was highest in synovial sarcoma (29/49, 59%) and lowest in uveal melanoma (5/81, 6%), NSCLC (3/103, 3%), and urothelial carcinoma (1/36, 3%), consistent with expected antigen positivity in each tumor type. Of the 49 patients who met pre-screening requirements, 30 proceeded to screening.

**Figure 1:**
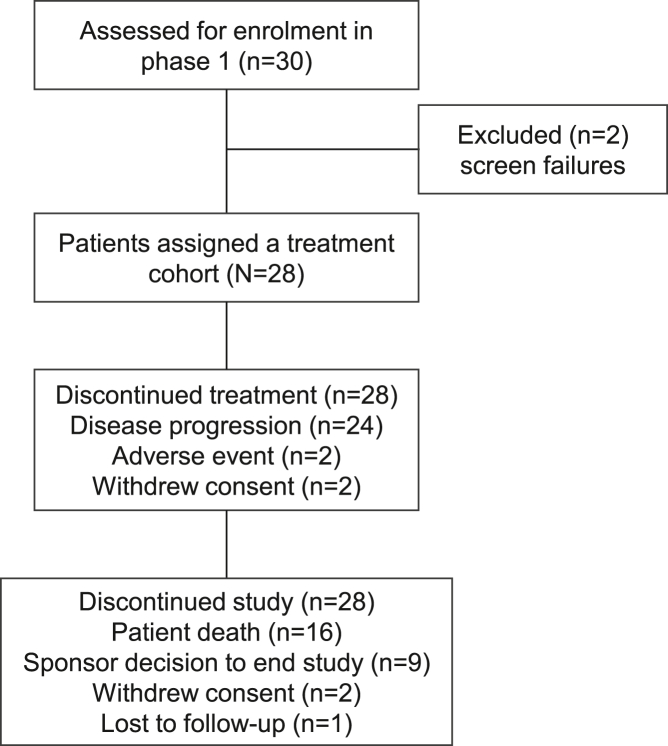
Disposition of participants (Consort diagram)

A total of 28 patients were enrolled from June 2018 to May 2021 and treated with IMCnyeso in 7 dose-escalation cohorts (Figure 1 and Table S1). Two patients did not meet eligibility criteria. All patients had baseline Eastern Cooperative Oncology Group performance status (ECOG PS) of 0 or 1 (Table 1). The study primarily enrolled patients with synovial sarcoma (*n* = 20), followed by melanoma (*n* = 7; 3 cutaneous, 3 uveal, and 1 mucosal). One patient with uveal melanoma was enrolled in a lower dose cohort (10 μg, duration 9.7 months) and subsequently re-enrolled in a higher dose cohort (30/100 μg). Patients with synovial sarcoma were heavily pre-treated; all received prior ifosfamide, 16/20 (80%) received prior anthracycline, and in the 100–300 μg cohorts, 6/9 (66%) received prior MAGE-A4 (*n* = 3) or NY-ESO-1 (*n* = 3) TCR-T treatment.

**Table 1:** Patient demographics and disease characteristics

	3–10 μg IMCnyeso (*n* = 7)	30 μg IMCnyeso (*n* = 5)	100–300 μg IMCnyeso (*n* = 17)	Total *N* = 28
**Age category (years), *n* (%)**				

18 to <50	4 (57%)	5 (100%)	6 (35%)	14 (50%)
50 to <65	3 (43%)	0	6 (35%)	9 (32%)
≥65	0	0	5 (29%)	5 (18%)

**Gender**				

Male, *n* (%)	3 (43%)	3 (60%)	10 (59%)	16 (57%)
Female, *n* (%)	4 (57%)	2 (40%)	7 (41%)	12 (43%)

**ECOG PS, *n* (%)**				

0	2 (29%)	2 (40%)	6 (35%)	9 (32%)
1	5 (71%)	3 (60%)	11 (65%)	19 (68%)

**Indication and prior treatment**				

Melanoma^a^	1 (14%)	0	7 (41%)	7 (25%)
Synovial sarcoma	6 (86%)	5 (100%)	9 (53%)	20 (71%)
Ifosfamide	6 (100%)	5 (100%)	9 (100%)	20 (100%)
Anthracycline	5 (83%)	4 (80%)	7 (78%)	16 (80%)
Pazopanib	3 (50%)	1 (20%)	3 (33%)	7 (35%)
TCR-T	1 (14%)	1 (20%)	6 (66%)	8 (40%)
Urothelial carcinoma	0	0	1 (6%)	1 (4%)

a One patient with uveal melanoma was sequentially enrolled in 2 cohorts: cohort 2 (IMCnyeso 10 μg) and cohort 5 (IMCnyeso 30/100 μg). Data are included in both cohort columns; however, only data from cohort 5 (IMCnyeso 30/100 μg) are summarized in the Total column.

### Exposure and disposition

The median duration of treatment was 1.8 months (range, 0.5–12.4 months), and the median dose intensity was 100%. Overall, 16 patients (57%) missed at least 1 weekly dose due to an adverse event (most frequent: chills, fatigue, decreased neutrophil count, and pyrexia; each *n* = 3 [10.7%]), and 3 patients received a reduced dose, or escalated more slowly to the target dose, due to an adverse event.

Disease progression was the most common reason for treatment discontinuation (*n* = 24; 86%) (Figure 1). Two patients discontinued treatment due to adverse events that were not related to study treatment (worsening dyspnea in a patient with synovial sarcoma who received a maximum dose of 100 μg [cohort 5] and progressing lung metastasis and hepatic failure in a patient with uveal melanoma and extensive liver metastasis at baseline who received a maximum dose of 180 μg [cohort 6]), and two patients withdrew consent. The most common reasons for study discontinuation were death due to disease progression (50%) and sponsor decision to end the study (32%).

### Safety

**Figure 2:**
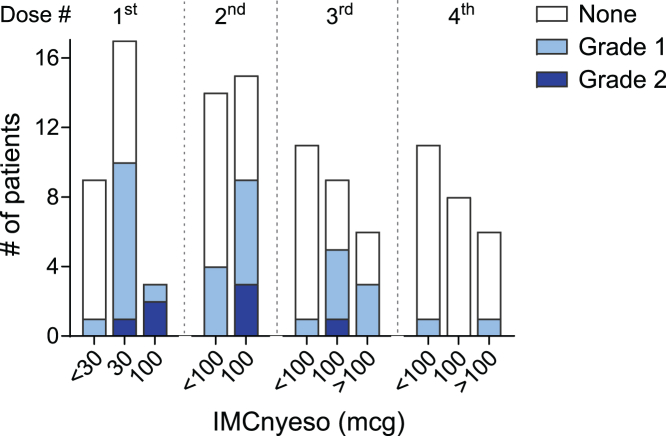
Incidence and severity of pyrexia Incidence and severity of pyrexia as a function of dose level, following the first 4 doses of IMCnyeso.

The most common treatment-related adverse events (TRAEs; ≥20% patients), pyrexia (*n* = 22; 79%), headache (*n* = 17; 61%), chills (*n* = 13; 46%), and cytokine release syndrome (CRS) (*n* = 12; 43%), were mild or moderate in severity and reversible with standard supportive care (Table 2). Most events occurred following the first three doses, with incidence and severity decreasing thereafter (Figure 2). The most common grade 3/grade 4 TRAEs were neutropenia/decreased neutrophil count (*n* = 4; 14%) and lymphopenia/decreased lymphocyte count (*n* = 3; 11%) (Table 2).

**Table 2:** Treatment-related adverse events (any grade incidence ≥20%)

Preferred term	3 μg (*n* = 4)	10 μg (*n* = 3)	30 μg (*n* = 5)	100 μg (*n* = 3)	30/100 μg (*n* = 5)	30/100/180 μg (*n* = 4)	30/100/300 μg (*n* = 5)	Total (*N* = 28) *n* (%)
**Any TRAE**	4	3	5	3	5	4	5	28 (100)
Pyrexia	1	1	4	3	5	3	5	22 (79)
Headache	2	2	4	3	2	0	3	17 (61)
Chills	0	1	3	2	2	3	3	13 (46)
CRS	0	0	0	2	2	3	5	12 (43)
Fatigue	2	1	1	2	0	2	1	9 (32)
Hypotension	0	1	0	0	1	1	3	6 (21)
**Any grade 3 or 4 TRAE**	0	0	0	2	1	8	10	9 (32)
Neutropenia, neutrophil count decreased	0	0	0	0	1	2	1	4 (14)
Lymphopenia, lymphocyte count decreased	0	0	0	2	0	1	0	3 (11)
Aspartate aminotransferase increased	0	0	0	0	0	0	1	1 (3.6)
Fatigue	0	0	0	0	0	1	0	1 (3.6)
Febrile neutropenia	0	0	0	0	0	0	1	1 (3.6)
Hypophosphatemia	0	0	0	0	0	1	0	1 (3.6)
Hypoxia	0	0	0	0	0	0	1	1 (3.6)
Sinus tachycardia	0	0	0	0	0	0	1	1 (3.6)
Leukopenia, white blood cell count decreased	0	0	0	0	0	1	0	1 (3.6)

Twelve patients (43%) experienced CRS per American Society for Transplantation and Cellular Therapy grading criteria. All events of CRS occurred in the setting where no premedications were given, were mild (grade 1) or moderate (grade 2) in severity, and occurred following an IMCnyeso dose ≥30 μg. Most events of CRS occurred following the first few doses and resolved on the day of onset; CRS did not occur beyond week 6. Four patients each experienced hypotension or hypoxia in conjunction with CRS. None of the CRS events included neurological symptoms consistent with immune effector cell-associated neurotoxicity syndrome. In most cases, CRS was managed symptomatically; two patients received tocilizumab (one patient had hypoxia and one had febrile neutropenia), and three patients received corticosteroid treatment (one patient had hypotension and two had hypoxia), with complete resolution in all cases.

No events of neutropenia/neutrophil count decrease were observed at doses <100 μg, while 5/17 patients who received doses of 100–300 μg had neutrophil counts that worsened to grade 3, suggesting a treatment-related change. Onset was typically 4–6 weeks after the first dose. In 4/5 cases, neutrophil counts recovered to normal within 1–2 weeks (Figure S1). Neutropenia was managed with dose interruption (4 patients) and growth factor support (2 patients) and did not recur following re-treatment with IMCnyeso.

No fatal adverse events were reported. Two dose-limiting toxicities occurred in cohort 7: a grade 3 febrile neutropenia following the 100 μg dose in the context of CRS, in a patient with a history of neutropenia, and a grade 4 aspartate aminotransferase increase following the 300 μg dose in a second patient, which rapidly resolved and did not recur with continued treatment at 300 μg.

### Pharmacokinetics and immunogenicity

**Figure 3:**
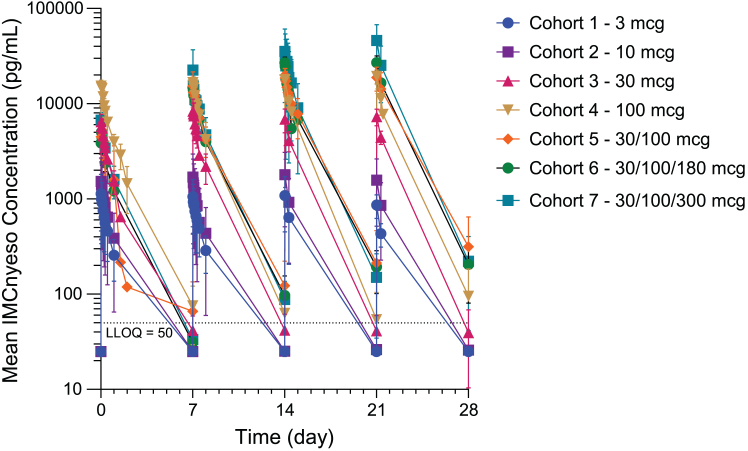
Pharmacokinetics IMCnyeso serum concentration (mean + SD for each cohort) versus time profiles following the first 4 weekly IV doses. See Table S1 for the number of participants in each cohort.

The exposure of IMCnyeso increased approximately in a dose-dependent manner (Figure 3). The half-life was approximately 25 h, and the steady-state volume of distribution indicated that the drug did not distribute extensively beyond the vasculature. Among 27 participants evaluable for anti-drug antibody, immunogenic responses to IMCnyeso were infrequent (2/27, 7%), and pharmacokinetics was affected (exposure reduced) in 1 participant (Table S2).

### Pharmacodynamics

**Figure 4:**
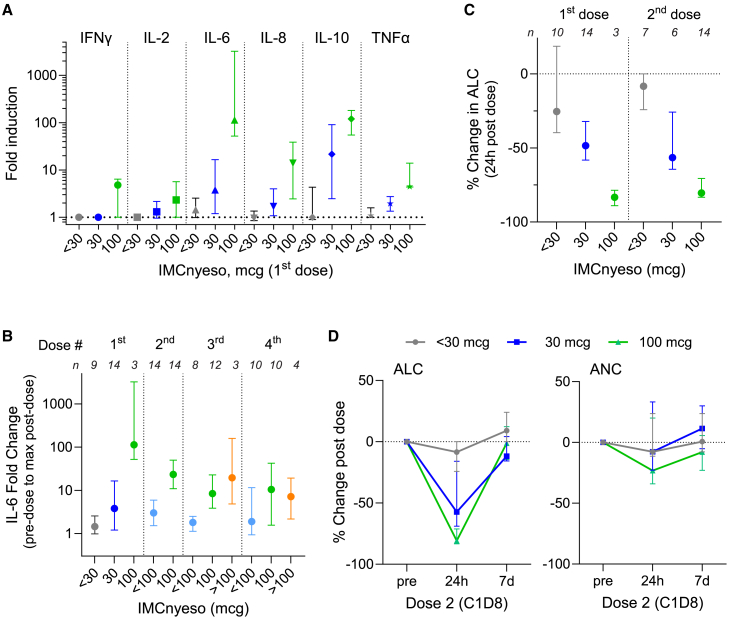
Pharmacodynamic changes with IMCnyeso (A) Induction of IFNγ, IL-2, IL-6, IL-8, IL-10, and TNF-α following the first dose of IMCnyeso, as a function of dose (*n* = 9, 14, and 3 at <30, 30, and 100 μg, respectively). Median and inter-quartile range (IQR) shown. (B) IL-6 induction as a function of dose level, following the first 4 doses of IMCnyeso. Median and interquartile range are shown. (C) Reduction in absolute lymphocyte counts following the first and second doses of IMCnyeso, as a function of dose. (D) Percent change in absolute lymphocyte count (ALC) and absolute neutrophil count (ANC) at 24 h and 7 days post second dose for patients receiving <30 (*n* = 7), 30 (*n* = 5), and 100 μg (*n* = 14) at dose 2. Median and IQR shown.

Dose-dependent induction of cytokines and transient decreases in lymphocyte count (consistent with T cell trafficking) were observed following the initial doses of IMCnyeso (Figure 4). Following the first dose, no significant cytokine induction was observed at doses of 3 or 10 μg; modest induction of interleukin (IL)-6 and IL-10 was observed at 30 μg (median 3.8 and 22-fold, respectively); and robust (>100-fold) induction of IL-6 and IL-10 was seen at 100 μg, with modest induction of IFNγ, IL-2, IL-8, and tumor necrosis factor alpha (TNF-α) (median 4.8, 2.3, 14, and 4.5-fold increase, respectively) (Figure 4A). Cytokine induction was attenuated after repeated dosing (Figure 4B). Transient decreases in lymphocyte were observed 1 day after the first and second doses, with moderate effect at 30 μg (median 38% decrease after the first dose, 59% after the second dose) and robust effect at 100 μg (median 83% decrease after the first dose, 81% after the second dose) (Figures 4C and 4D). No on-treatment biopsies were collected.

### Antitumor activity

**Figure 5:**
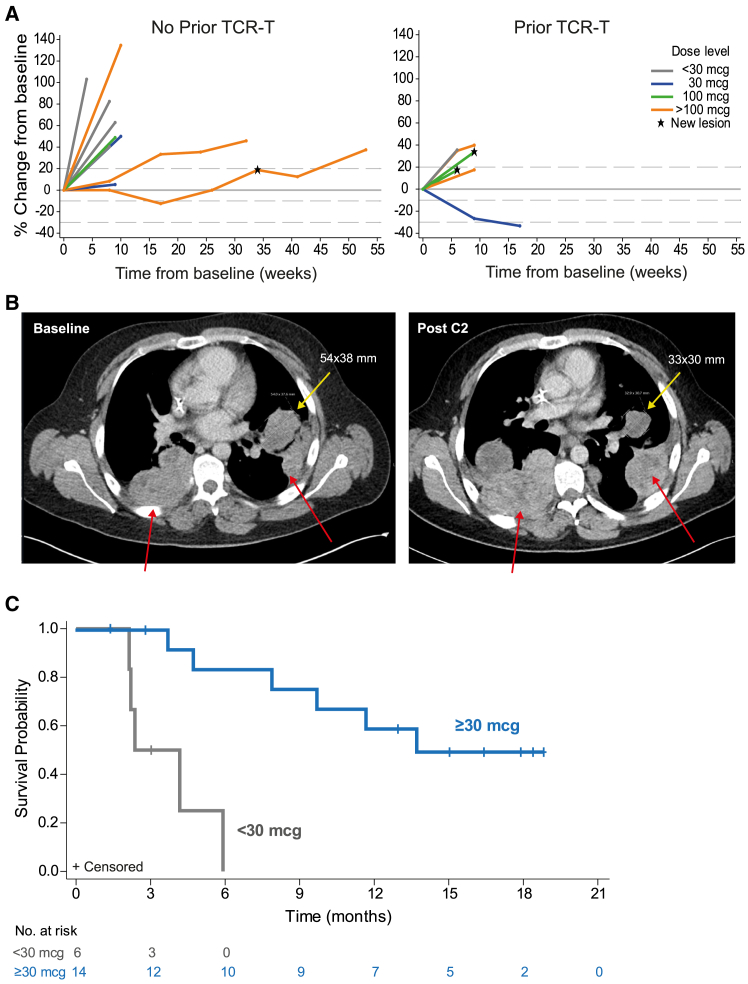
Efficacy outcomes (A) Change in sum of target lesion diameters for evaluable patients with synovial sarcoma who had (*n* = 7) or had not (*n* = 11) received prior TCR-T therapy by dose group (gray: <30 μg, blue: 30 μg, green: 100 μg, orange: >100 μg). Two response-evaluable patients are not shown; one had non-target lesions only (Best overall response [BOR] of stable disease) and one missed pre-treatment scans (BOR progressive disease). (B) Baseline and on-treatment scans for a patient (30 μg cohort) with mixed response (reduction in the lesion marked in yellow but increase in the lesions marked in red). (C) Kaplan-Meier plot of overall survival for patients who received <30 μg IMCnyeso versus those who received ≥30 μg IMCnyeso.

No confirmed responses per RECIST v.1.1 were observed. Changes in tumor burden as a function of time are shown for evaluable patients with synovial sarcoma (*n* = 18) who did (*n* = 7) or did not (*n* = 11) receive prior TCR-T therapy in Figure 5A. Of these patients, all who received subtherapeutic doses (3–10 μg; *n* = 5) progressed rapidly. At pharmacologically active doses (30–300 μg), 4 (29%) had a best response of stable disease, 9 (64%) had a best response of progressive disease, and 1 was non-evaluable. Two patients at 30 μg experienced mixed responses, with shrinkage of some lesions but growth in others (example shown in Figure 5B and Table S3). Median OS was approximately 3 months at doses of 3–10 μg and approximately 12 months at doses of 30–300 μg (Figure 5C).

Among 7 patients with melanoma, one (14%, in the 30/100/300 μg cohort) had a best response of stable disease, 5 (71%) had a best response of progressive disease, and 1 was non-evaluable, and the one patient with urothelial carcinoma progressed at the first scan.

## Discussion

IMCnyeso is an ImmTAC bispecific T cell engager targeting NY-ESO-1/LAGE-1A-positive tumors. As with tebentafusp, pharmacodynamic changes following IMCnyeso, namely induction of pro-inflammatory cytokines and transient decreases in lymphocytes, are consistent with a mechanism of T cell activation. These changes were observed at doses of 30 μg and higher.

IMCnyeso was generally well tolerated, with mild-to-moderate CRS and associated signs/symptoms as the most common adverse events. As with tebentafusp, CRS was most common with the initial doses and attenuated with continued treatment. To mitigate CRS, a step-up dosing regimen was implemented for the higher dose cohorts. Neutropenia was an emergent adverse event noted at higher (≥100 μg) doses of IMCnyeso, which in some cases resolved with treatment interruption alone and without the need for granulocyte colony stimulating factor support. Interestingly, neutropenia was not observed with tebentafusp treatment, which may be related to either the lower recommended dose of tebentafusp (68 μg) or the target population, which is not heavily pretreated with myelotoxic chemotherapies (uveal melanoma compared to synovial sarcoma). Enrollment for the potential recommended dose levels of 180 and 300 μg was not completed at the time the sponsor decided to discontinue development for strategic reasons related to low enrollment across tumor types and not due to any safety issues. Consequently, the maximum tolerated dose (MTD) was not fully characterized.

Efficacy could not be fully characterized due to difficulty in enrolling patients with synovial sarcoma who had not received prior TCR-T therapy as these types of agents may impact response to a subsequent cancer testis antigen/NY-ESO-1-targeted immunotherapy as investigated here. However, the sharp contrast in OS observed for patients treated at pharmacodynamically active doses (≥30 μg) versus those who were not suggests that this molecule may have clinical activity. It is also worth noting that the median OS of 12 months observed for this group of patients with synovial sarcoma is highly similar to that reported for the TCR-T cell therapy afami-cel (formerly ADP-A2M4) in a similar small cohort of patients with synovial sarcoma in a phase 1 trial (median OS: 13.3 months).^12^ Given that the majority of patients in this study had progressed on prior TCR-T cell therapies, these data, although immature, suggest that clinical benefit may have been observed in these patients with longer follow-up. However, conclusions about efficacy cannot be derived due to the limited sample size, early termination of the study (prior to completion of dose escalation) due to strategic reason, and immaturity of the data.

### Limitations of the study

The main limitation of this phase 1 study was the small number of patients, primarily due to the low pre-screening success (<10%) in uveal melanoma, NSCLC, and urothelial carcinoma, which contributed to the early termination of the study and the short duration of follow-up. Other limitations include the heterogeneous patient population and lack of on-treatment tumor biopsies.

### Conclusion

IMCnyeso doses of 30–300 μg showed an acceptable safety profile and pharmacodynamic activity. The MTD and RP2D were not fully characterized as the study was ended early for strategic reasons. IMCnyeso may have limited clinical utility, as its target antigen is rarely present in NSCLC and urothelial carcinoma, and TCR-T is already in pivotal trials in synovial sarcoma. Related ImmTAC molecules targeting MAGE-A4 and preferentially expressed antigen in melanoma (PRAME), cancer-testis antigens present in a broader range of malignancies, have demonstrated durable responses in early-stage clinical trials.^13^^,^^14^

## Resource availability

### Lead contact

All requests for additional information and resources should be directed to the lead contact, Juanita S. Lopez (juanita.lopez@icr.ac.uk) and the sponsor Immunocore Ltd (info@immunocore.com).

### Materials availability

This study did not generate new unique reagents.

### Data and code availability

• De-identified participant data used in these analyses can be requested from the lead contact.
• This manuscript does not report original code.
• Any additional information required to reanalyze the data reported in this work are available from the lead contact upon request.

## Acknowledgments

The authors would like to thank all participating patients, their families, investigators and sub-investigators, and staff at the study sites. This study was funded by Immunocore Ltd.

## Author contributions

J.S.L., J.W., S.M., and J.R. contributed to the conception, design, and planning of the study. All authors contributed to the acquisition, analysis, and interpretation of the data. R.E., J.W., and S.M. performed or oversaw statistical analyses. J.S.L., M.M., M.O.B., F.T., B.A.V.T., S.P.D., M.L.J., T.S., H.-T.A., and J.R. contributed to provision of study materials or patients. S.M. drafted the manuscript. All authors had access to the data, critically reviewed iterations of the manuscript, and approved the final draft for submission.

## Declaration of interests

J.S.L. discloses consulting fees for participation in an Advisory Board for Roche Genentech, GSK, Basilea, and Pierre Faber.

M.O.B. discloses consultant/Advisory Board: Adaptimmune, Bristol Myers Squibb Canada, GlaxoSmithKline, Immunocore, Instil Bio, Iovance Biotherapeutics, Merck, Novartis, Pfizer, Sanofi Pasteur Inc., Sun Pharma, IDEAYA Biosciences, Medison, and Regeneron; safety review committee: GlaxoSmithKline and Adaptimmune; research funding: 10.13039/100004334Merck, 10.13039/100031835Takara Bio, and 10.13039/100004336Novartis.

F.T. discloses consulting or advisory role: T-knife, Immatics, GuidePoint Pharmacy, Leucid Bio, Scenic Biotech, CytomX Therapeutics, Grey Wolf Therapeutics, AstraZeneca, and OncoBayes; research funding: 10.13039/100004319Pfizer (Inst), Genmab (Inst), Novartis (Inst), AstraZeneca (Inst), CytomX Therapeutics (Inst), Janssen (Inst), Takeda (Inst), Adaptimmune (Inst), Bristol Myers Squibb (Inst), 10.13039/100004330GlaxoSmithKline (Inst), 10.13039/100004337Roche (Inst), 10.13039/100006483AbbVie (Inst), 10.13039/100031787Immunocore (Inst), Achilles Therapeutics (Inst), 10.13039/100010795Chugai (Inst), RS Oncology (Inst), Crescendo Biologics (Inst), Oxford VacMedix (Inst), 10.13039/100004339Sanofi (Inst), Novalgen (Inst), 10.13039/100018536NuCana (Inst), 10.13039/100017655Incyte (Inst), and T-knife (Inst); travel, accommodations, expenses: Scenic Biotech.

B.A.V.T. discloses research grants from Polaris, royalties or licenses from Accuronix Therapeutics for Sigma-2 Receptor Ligands and Therapeutic Uses Thereof (006766), Modular Platform for Targeted Therapeutic Delivery (006755) and Sigma-2 Receptor Ligand Drug Conjugates as Antitumor Compounds, Methods of Synthesis and Uses Thereof (014229), consulting fees from Deciphera Pharmaceuticals, Daiichi Sankyo Inc., EcoR1, Advenchen, Putnam, Salarius Pharmaceuticals, Boxer Capital, Acuta Capital Partners, LLC, AADI, Hinge Bio, Kronos Bio, CRISPR Therapeutics, and Galapagos, payment or honoraria from Iterion Therapeutics, Total Health Conference, Oncology Education, and Beijing Biostart Pharmaceuticals Co., expert legal testimony payment from Arnold Todaro Welch & Foliano, Phelan Tucker Law LLP, and Anderson & Reynolds PLC, travel support for attending meetings and/or travel from Krones, Polaris, and Adaptimmune Ltd., participation on advisory boards for Apexigen Inc., Daiichi Sankyo, Deciphera Pharmaceuticals, Inc., Bayer, PCT Therapeutics, Aadi Bioscience, Boehringer Ingelheim, Agenus, Regeneron Pharmaceuticals, Advenchen, Curtis, and Syneos Health, as well as a leadership or a fiduciary role for Polaris (not paid).

S.P.D. discloses institutional research funding from 10.13039/100002429Amgen, Bristol Meyers Squibb, Deciphera, 10.13039/100004755EMD Serono, Incyte, Merck, and Nektar Therapeutics, has served as a consultant or on advisory boards for Adaptimmune, Amgen, EMD Serono, GlaxoSmithKline, Immune Design, Immunocore, Incyte, Merck, Nektar Therapeutics, Pfizer, Servier, and Rain Therapeutics, and has served on data safety monitoring boards for Adaptimmune, GlaxoSmithKline, Merck, and Nektar Therapeutics.

M.L.J. discloses grants from AbbVie, Amgen, Apexigen, Arcus Biosciences, 10.13039/100007174Array BioPharma, Artios Pharma, 10.13039/100004325AstraZeneca, Atreca, 10.13039/100017239BeiGene, 10.13039/100022747BerGenBio, 10.13039/100001003Boehringer Ingelheim, 10.13039/100017658Calithera Biosciences, Checkpoint Therapeutics, Corvus Pharmaceuticals, Curis, CytomX, 10.13039/501100022274Daiichi Sankyo, Dracen Pharmaceuticals, Dynavax, Lilly, 10.13039/100004755EMD Serono, 10.13039/100004328Genentech-Roche, Genmab, Genocea Biosciences, GlaxoSmithKline, Gritstone Oncology, 10.13039/100031949Guardant Health, Harpoon, Hengrui Therapeutics, Immunocore, Incyte, Janssen, 10.13039/100016765Jounce Therapeutics, Kadmon Pharmaceuticals, 10.13039/100016936Loxo Oncology, Lycera, Merck, 10.13039/100016957Mirati Therapeutics, Neovia Oncology, Novartis, OncoMed Pharmaceuticals, Pfizer, PMV Pharmaceuticals, 10.13039/100009857Regeneron Pharmaceuticals, 10.13039/100018287Ribon Therapeutics, Sanofi, Seven and Eight Biopharmaceuticals-Birdie Biopharmaceuticals, Shattuck Labs, 10.13039/100020568Silicon Therapeutics, Stem CentRx, 10.13039/100018201Syndax Pharmaceuticals, Takeda Pharmaceuticals, Tarveda, TCR2 Therapeutics, TMUNITY Therapeutics, 10.13039/100007270University of Michigan, and WindMIL and institutional fees for consulting services from AbbVie, Amgen, AstraZeneca, Atreca, Boehringer Ingelheim, Calithera Biosciences, Checkpoint Therapeutics, CytomX, Daiichi Sankyo, EMD Serono, Genentech-Roche, GlaxoSmithKline, Gritstone Oncology, Guardant Health, Incyte, Janssen, Loxo Oncology, Merck, Mirati Therapeutics, Novartis, Pfizer, Ribon Therapeutics, Sanofi, WindMIL, Achilles Therapeutics, Bristol Myers Squibb, Editas Medicine, Eisai, G1 Therapeutics, IDEAYA Biosciences, Lilly, and Association of Community Cancer Centers.

T.S. discloses advisory/consulting: Immunocore and Castle Biosciences; research funding to institution: Immunocore and Verastem.

H.-T.A. discloses other support from 10.13039/100016646HCA Healthcare UK during the conduct of the study, grants and other support from Bicycle, grants and personal fees from BeiGene, personal fees from Guardant, Servier, Bayer, Labgenius, CellCentric, Engitix, and iOnctura, and grants from Taiho outside the submitted work.

R.E. was an employee of Immunocore.

J.W. and S.M. are employees and holder of stocks/stock options of Immunocore.

J.R. discloses consulting or advisory role: Peptomyc, Kelun Pharmaceuticals/Klus Pharma, Ellipses Pharma, Molecular Partners, and iOnctura; research funding: Blueprint Medicines (Inst), Black Diamond Therapeutics (Inst), 10.13039/100009947Merck Sharp & Dohme (Inst), Hummingbird (Inst), Yingli Pharma (Inst), Vall d'Hebron Institute of Oncology/Cancer Core Europe (Inst), Novartis (Inst), 10.13039/100006399Spectrum Pharmaceuticals (Inst), 10.13039/501100018688Symphogen (Inst), BioAtla (Inst), Pfizer (Inst), Genmab (Inst), CytomX Therapeutics (Inst), Kelun (Inst), Takeda/Millennium (Inst), GlaxoSmithKline (Inst), 10.13039/100009954Taiho Pharmaceutical (Inst), Roche (Inst), Bicycle Therapeutics (Inst), Merus (Inst), Curis (Inst), 10.13039/100004326Bayer (Inst), AADi (Inst), Nuvation Bio (Inst), Fore Biotherapeutics (Inst), BioMed Valley Discoveries (Inst), Loxo (Inst), Hutchison MediPharma (Inst), 10.13039/100031793Cellestia Biotech (Inst), Deciphera (Inst), IDEAYA Biosciences (Inst), Amgen (Inst), Tango Therapeutics (Inst), Mirati Therapeutics (Inst), and Linnaeus Therapeutics (Inst); travel, accommodations, expenses: ESMO; other relationship: Vall d'Hebron Institute of Oncology/Ministerio De Empleo Y Seguridad Social, Chinese University of Hong Kong, Boxer Capital, and Tang Advisors.

## STAR★Methods

### Key resources table

**Table:** 

REAGENT or RESOURCE	SOURCE	IDENTIFIER
**Biological samples**		

Peripheral blood serum samples	Participating study sites	N/A

**Chemicals, peptides, and recombinant proteins**		

IMCnyeso	Immunocore Ltd	Patent No.: US 11,639,374 B2

**Critical commercial assays**		

HLA typing	American Red Cross Histocompatibility Laboratory Services	N/A
Custom *therascreen*® NYESO RGQ RT-PCR kit	Qiagen	N/A
Roto-Gene Q MDx	Qiagen	9002032

**Software and algorithms**		

SAS® software version 9.4	SAS Institute Inc	RRID: SCR_008567
GraphPad Prism® version 10	GraphPad Software LLC	RRID: SCR_002798
Phoenix® WinNonlin® Version 8.1	Certara, Princeton, New Jersey	N/A

**Other**		

Clinical trial registration number	https://clinicaltrials.gov/	NCT03515551

### Experimental model and study participant details

This international, multi-center study included human participants. The study was approved by the Institutional Review Board/Independent Ethics Committee of each study site and followed the Declaration of Helsinki and International Conference on Harmonisation Good Clinical Practice guidelines. All patients provided written informed consent before any study procedures were performed, with separate consents for Pre-Screening and Main Study. HLA-A∗02:01-positive adults, both male and female, with advanced NY-ESO-1 and/or LAGE-1A-positive advanced malignancies were enrolled. Demographic information, including age and gender, was provided in Table 1.

### Method details

#### Study design

Study IMCnyeso-101 was an open-label, multi-center, phase 1/2 trial of IMCnyeso in patients with advanced malignancies. The primary objective of this study was to determine the maximum tolerated dose (MTD) and/or recommended Phase 2 dose (RP2D). Additional objectives included anti-tumor activity, pharmacokinetics, immunogenicity, and pharmacodynamic changes. Cohorts enrolled 3 to 6 patients. Dose escalation decisions were made following review of all available (safety, pharmacokinetic, pharmacodynamic, and efficacy data), were guided by a Bayesian logistic regression model (BLRM) and were subject to both the escalation with overdose control (EWOC) principle and the escalation rule.

#### Study population

Study IMCnyeso-101 enrolled HLA-A∗02:01+ patients aged ≥18 years, having Eastern Cooperative Oncology Group performance status 0 or 1, with advanced NY-ESO-1 and/or LAGE-1A positive-NSCLC, melanoma, urothelial carcinoma, or synovial sarcoma relapsed from, refractory to, or intolerant to standard therapies. Patients previously treated with T cell therapies were permitted to enroll.

#### Treatment protocol

Patients received IMCnyeso by weekly intravenous infusion until unacceptable toxicity, disease progression, or other reason to discontinue. Treatment beyond initial radiographic progression was allowed in the absence of clinically significant progression (e.g., decline in performance status, threat to vital organ). Dosing began at 3 μg, which was predicted to provide a maximum serum concentration equal to the minimum anticipated biological effect level (MABEL). In the first 4 cohorts (3, 10, 30, or 100 μg), no step-up-dosing regimen was used, and patients received the same dosage of IMCnyeso each week (Table S1). Starting with Cohort 5, a step-up-dosing regimen was implemented (30 μg on Day 1, then target dose of 100 μg weekly starting on Day 8) to mitigate the risk of cytokine-mediated AEs.^15^^,^^16^ For Cohort 6 and Cohort 7, an additional step dose (100 μg) was incorporated on Day 8 and the target dose (180 μg in Cohort 6 and 300 μg in Cohort 7) was given on Day 15 onwards.

#### Endpoints and assessments

During pre-screening, patients underwent HLA-A allele typing (American Red Cross Histocompatibility Laboratory Services) and tumor expression of NY-ESO-1 and LAGE-1A was assessed using a custom validated therascreen NYESO RGQ RT-PCR kit assay run on the Roto-Gene Q MDx platform (Qiagen) in a CAP/CLIA accredited laboratory (MolecularMD). Patients were required to be HLA-A∗02:01-positive and have an NY-ESO-1 and/or LAGE-1A positive tumor prior to entering Screening.

Extended (overnight) monitoring was required after the first 2 (fixed dosing cohorts 1–4) or 3 (step-up dosing cohorts 5–7) doses. Premedications were not permitted prior to the first dose. Cytokine release syndrome was graded by the investigators per 2019 recommendations of the American Society for Transplantation and Cellular Therapy (ASTCT)^17^; all other adverse events were graded according to the National Cancer Institute Common Terminology Criteria for Adverse Events (NCI CTCAE) version 4.03. Cytokine release syndrome was defined as an adverse event of special interest (AESI); therefore, the severity and timing of associated signs and symptoms (including pyrexia, hypoxia, and hypotension) were also collected. A 28-day dose limiting toxicity (DLT) period was used for all schedules. DLTs included Grade ≥3 AE occurring during the DLT evaluation period, with a suspected relationship to study drug, with limited modifications.

Antitumor activity was assessed by tumor measurements (CT or MRI) performed during screening, every 8 weeks for 40 weeks, then every 12 weeks thereafter. Disease response was evaluated by study investigators per RECIST v1.1.^18^ Overall survival was measured from the start of treatment to the time of death. Patients who did not die were censored on the last date on which they were known to be alive.

Serum samples were collected for PK profiling for the first 4 doses at the following timepoints: for the first two infusions (C1D1 and C1D8), samples were collected pre-dose (within 2 h), at end of infusion (EOI), and at 1h, 2h, 4h, 6h, 8h, 12h and 24h post-EOI with optional samples at 36h and 48h post-EOI following C1D1; for the 3^rd^ and 4^th^ infusions (C1D15 and C1D22), samples were collected pre-dose, at EOI, and at 8h and 12h (optional) post-EOI; and following the first cycle, pre-dose and EOI samples were collected for C2D1, C2D15, C3D1 and D1 of subsequent odd-numbered cycles. Serum concentrations of IMCnyeso were determined using an electrochemiluminescent immunoassay (ECLIA). Non-compartmental analysis (NCA) was performed on IMCnyeso concentration-time data using Phoenix WinNonlin (Version 8.1, Certara, Princeton, New Jersey). Each sample assayed in duplicate with a re-test performed in duplicate if the initial test variation was >20%.

Serum samples were collected periodically for anti-drug antibody (ADA) testing. Anti-IMCnyeso antibodies were detected using a bridging format ECLIA in a tiered analysis whereby samples testing positive in the screening assay were then evaluated in confirmatory and titer assays.

Serum samples were collected for cytokine testing before and at multiple timepoints following the first 2 doses (fixed-dose regimen, Cohorts 1–4) or first 3 doses (step-dose regimen, Cohorts 5–7). Concentrations of interleukin-2 (IL-2), IL-6, IL-8, IL-10, tumor necrosis factor alpha (TNFα) and interferon-gamma (IFNγ) were determined at the study central laboratory (PPD) using a Luminex method (R&D Systems, Minneapolis, Minnesota). Concentrations were calculated based on the average of duplicate readings for each sample.

### Quantification and statistical analysis

Dose-escalation decisions were informed by Bayesian logistic regression model with overdose control (EWOC). Adverse events were monitored for all patients who received at least 1 dose of the study treatment. Preliminary efficacy was assessed in evaluable patients with an original diagnosis of synovial sarcoma. Descriptive statistics were provided for patient demographics and characteristics, adverse events, pharmacokinetics and pharmacodynamics. Overall survival was estimated using Kaplan-Meier methods. All clinical data analyses, summaries, and outputs were produced using either SAS version 9.4. Pharmacokinetic and pharmacodynamic summary plots were produced using GraphPad Prism version 10.

### Additional resources

This study has been registered on “https://clinicaltrials.gov/,” ID: NCT03515551.

## Supplemental information

Document S1. Figure S1 and Tables S1–S3

Document S2. Article plus supplemental information

## References

[1] Cebon J.S., Gore M., Thompson J.F., Davis I.D., McArthur G.A., Walpole E., Smithers M., Cerundolo V., Dunbar P.R., MacGregor D. (2020). Results of a randomized, double-blind phase II clinical trial of NY-ESO-1 vaccine with ISCOMATRIX adjuvant versus ISCOMATRIX alone in participants with high-risk resected melanoma. J. Immunother. Cancer.

[2] Thomas R., Al-Khadairi G., Roelands J., Hendrickx W., Dermime S., Bedognetti D., Decock J. (2018). NY-ESO-1 Based Immunotherapy of Cancer: Current Perspectives. Front. Immunol..

[3] D’Angelo S., Demetri G., Tine B.V., Druta M., Glod J., Chow W., Pandya N., Hasan A., Chiou V., Tress J. (2020). Regul. young Investig. award Abstr.

[4] D’Angelo S.P., Araujo D.M., Abdul Razak A.R., Agulnik M., Attia S., Blay J.-Y., Carrasco Garcia I., Charlson J.A., Choy E., Demetri G.D. (2024). Afamitresgene autoleucel for advanced synovial sarcoma and myxoid round cell liposarcoma (SPEARHEAD-1): an international, open-label, phase 2 trial. Lancet.

[5] Lowe K.L., Cole D., Kenefeck R., OKelly I., Lepore M., Jakobsen B.K. (2019). Novel TCR-based biologics: mobilising T cells to warm “cold” tumours. Cancer Treat Rev..

[6] Liddy N., Bossi G., Adams K.J., Lissina A., Mahon T.M., Hassan N.J., Gavarret J., Bianchi F.C., Pumphrey N.J., Ladell K. (2012). Monoclonal TCR-redirected tumor cell killing. Nat. Med..

[7] Watson A.G., Britton-Rivet C., Stanhope S., Collins L., Ranade K., Benlahrech A. (2022). Regul Young Investigator Award Abstr.

[8] Bethune M.T., Li X.-H., Yu J., McLaughlin J., Cheng D., Mathis C., Moreno B.H., Woods K., Knights A.J., Garcia-Diaz A. (2018). Isolation and characterization of NY-ESO-1–specific T cell receptors restricted on various MHC molecules. Proc. Natl. Acad. Sci..

[9] de Carvalho F., Vettore A.L., Inaoka R.J., Karia B., Andrade V.C.C., Gnjatic S., Jungbluth A.A., Colleoni G.W.B. (2010). Evaluation of LAGE-1 and NY-ESO-1 expression in multiple myeloma patients to explore possible benefits of their homology for immunotherapy. Cancer Immun.

[10] McCormack E., Adams K.J., Hassan N.J., Kotian A., Lissin N.M., Sami M., Mujić M., Osdal T., Gjertsen B.T., Baker D. (2013). Bi-specific TCR-anti CD3 redirected T-cell targeting of NY-ESO-1- and LAGE-1-positive tumors. Cancer Immunol. Immunother..

[11] Nathan P., Hassel J.C., Rutkowski P., Baurain J.-F., Butler M.O., Schlaak M., Sullivan R.J., Ochsenreither S., Dummer R., Kirkwood J.M. (2021). Overall Survival Benefit with Tebentafusp in Metastatic Uveal Melanoma. N. Engl. J. Med..

[12] Hong D.S., Tine B.A.V., Biswas S., McAlpine C., Johnson M.L., Olszanski A.J., Clarke J.M., Araujo D., Blumenschein G.R., Kebriaei P. (2023). Autologous T cell therapy for MAGE-A4+ solid cancers in HLA-A∗02+ patients: a phase 1 trial. Nat Med.

[13] Blumenschein G.R., Davar D., Gutierrez R., Segal N.H., Johnson M.L., Dar M.M., Marshall S. (2020). A phase I/II first-in-human study of a novel anti-MAGE-A4 TCR/anti-CD3 bispecific (IMC-C103C) as monotherapy and in combination with atezolizumab in HLA-A∗02:01-positive patients with MAGE-A4-positive advanced solid tumors (IMC-C103C-101). J. Clin. Oncol..

[14] Hamid O., Sato T., Davar D., Callahan M.K., Thistlethwaite F., Aljumaily R., Johnson M.L., Arkenau H.-T., Ileana Dumbrava E., Izar B. (2022). 728O Results from phase I dose escalation of IMC-F106C, the first PRAME × CD3 ImmTAC bispecific protein in solid tumors. Ann. Oncol..

[15] Saber H., Del Valle P., Ricks T.K., Leighton J.K. (2017). An FDA oncology analysis of CD3 bispecific constructs and first-in-human dose selection. Regul. Toxicol. Pharmacol..

[16] Carvajal R.D., Nathan P., Sacco J.J., Orloff M., Hernandez-Aya L.F., Yang J., Luke J.J., Butler M.O., Stanhope S., Collins L. (2022). Phase I Study of Safety, Tolerability, and Efficacy of Tebentafusp Using a Step-Up Dosing Regimen and Expansion in Patients With Metastatic Uveal Melanoma. J. Clin. Oncol..

[17] Lee D.W., Santomasso B.D., Locke F.L., Ghobadi A., Turtle C.J., Brudno J.N., Maus M.V., Park J.H., Mead E., Pavletic S. (2019). ASTCT Consensus Grading for Cytokine Release Syndrome and Neurologic Toxicity Associated with Immune Effector Cells. Biol. Blood Marrow Transplant..

[18] Eisenhauer E.A., Therasse P., Bogaerts J., Schwartz L.H., Sargent D., Ford R., Dancey J., Arbuck S., Gwyther S., Mooney M. (2009). New response evaluation criteria in solid tumours: Revised RECIST guideline (version 1.1). Eur. J. Cancer.

