# Supplementary material for: Phase 1 study of IMCnyeso, a T cell receptor bispecific ImmTAC targeting NY-ESO-1-expressing malignancies
Source: Cell Rep Med. 2025 Mar 6;6(4):101994. doi: 10.1016/j.xcrm.2025.101994 (PMC12047507; doi:10.1016/j.xcrm.2025.101994)
Supplement: Document S1. Figure S1 and Tables S1–S3 [file mmc1.pdf]

Cell Reports Medicine, Volume *6*

Supplemental information

Phase 1 study of IMCnyeso, a T cell receptor bispecific ImmTAC targeting NY-ESO-1-expressing malignancies

Juanita S. Lopez, Mohammed Milhem, Marcus O. Butler, Fiona Thistlethwaite, Brian A. Van Tine, Sandra P. D'Angelo, Melissa L. Johnson, Takami Sato, Hendrik-Tobias Arkenau, Ramakrishna Edukulla, Jason Wustner, Shannon Marshall, and Jordi Rodon

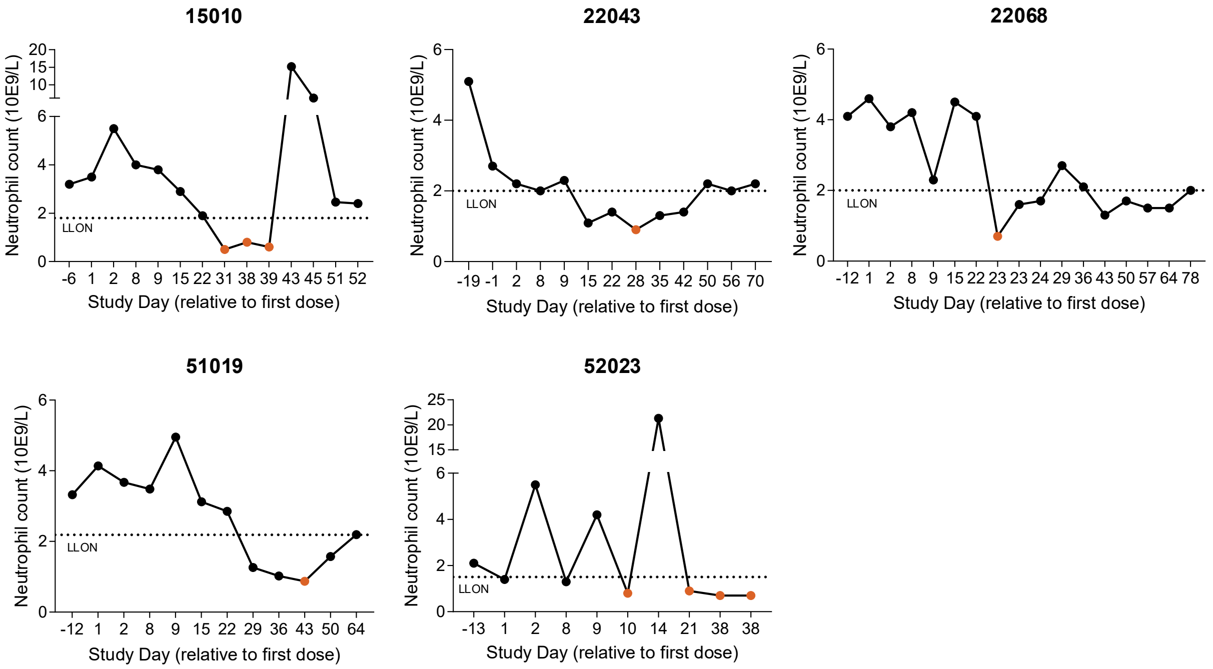

Figure S1. ANC for patients with Grade 3 neutropenia/neutrophil count decrease. Related to Table 3. Absolute neutrophil counts by study day number relative to first dose of IMCnyeso for patients who experienced grade 3 adverse event of neutropenia or neutrophil count decrease. Dotted line represents the lower limit of normal (LLON) and orange symbol denotes grade 3 adverse event.

Table S1. Dose escalation schema. Related to Figure 1 and STAR Methods

Cohort	# Patients	Tumor types	C1D1 dose	C1D8 dose	≥C1D15 dose
1	4	4 SynS	3 mcg	3 mcg	3 mcg
2	3	2 SynS, 1 UM [a]	10 mcg	10 mcg	10 mcg
3	5	5 SynS	30 mcg	30 mcg	30 mcg
4	3	1 MM, 2 SynS	100 mcg	100 mcg	100 mcg
5	5	3 SynS, 1 UC, 1 UM [a]	30 mcg	100 mcg	100 mcg
6	4	2 SynS, 2 UM	30 mcg	100 mcg	180 mcg
7	5	3 CM, 2 SynS	30 mcg	100 mcg [b]	300 mcg [b]

CM = cutaneous melanoma; DLT = dose-limiting toxicity; MM = mucosal melanoma; SynS = synovial sarcoma; UC = urothelial carcinoma; UM = uveal melanoma.

[a] One patient in Cohort 2 subsequently re-enrolled in Cohort 5. This patient is included in both rows above and included in the total counts as one unique individual.
[b] Two patients in Cohort 7 experienced DLT, one following the 100 mcg dose on C1D8 and one following a dose of 300 mcg on C1D22:

- A DLT of Grade 3 febrile neutropenia was reported in a patient with metastatic synovial sarcoma, with Grade 2 neutropenia prior to the first dose (1.4×10^9/L). Following the second dose, the patient developed Grade 2 CRS with sinus tachycardia (heart rate 100 to 120 bpm) starting 2 hours after end of infusion and pyrexia (39ºC) at 32 hours after the end of infusion; Grade 3 neutropenia was observed on Day 10. Fever and neutropenia resolved the same day, following treatment with paracetamol, ibuprofen, tocilizumab, and peg-filgrastim. The patient missed the next 4 doses due to headache, recurrent neutropenia, and refusal to attend clinic and subsequently withdrew consent.
- A DLT of Grade 4 aspartate aminotransferase (AST) increased following the first dose of 300 mcg was reported in a patient with metastatic cutaneous melanoma. This patient had liver metastasis and normal liver function tests at baseline. One day after the second dose (100 mcg), the patient was found to have an asymptomatic Grade 4 AST increase (22.2 × ULN) which improved to 5.6 × ULN in less than 24 hours (therefore not meeting DLT criteria). The patient received 100 mcg again at the third dose and experienced a transient Grade 1 AST increase. On Day 22, the patient escalated to the target dose of 300 mcg and was found to have an asymptomatic Grade 4 AST increase the next day (38.1 × ULN) which improved to 9.6 × ULN on Day 24 and fully resolved by Day 29. As AST was >8 × ULN and confirmed by re-testing after more than 24 hours, this second event met DLT criteria. The patient continued treatment for another 3 months with a best response of stable disease and then discontinued because of progressive disease.

Table S2. Immunogenicity. Related to Figure 2.

Category	Number of participants
Evaluable for ADA	27
ADA induced or boosted during treatment	2/27 (7.4%)
ADA Induced	1/27 (3.7%)
ADA Boosted	1/27 (3.7%)
ADA detected at baseline with no meaningful change in titer during treatment	2 (7.4%)

Boosted: ADA positive at baseline and post-baseline, with increase in titer during treatment. Induced: ADA negative at baseline and positive post-baseline.

Table S3. Details for patients with synovial sarcoma, IMCnyeso dose ≥ 30 mcg. Related to Figure 4.

Age category / Baseline ECOG PS	Prior Systemic Cancer Therapies	IMCnyeso Treatment	Response	Subsequent Cancer Treatment	Survival
18 -<50 ECOG PS 0	Doxorubicin/ifosfamide, NY-ESO-1 T cells, pazopanib, olaratumumab, doxorubicin	30 mcg On treatment 3.9 months	BOR SD, PD at Day 118 with tumor shrinkage (-33%)	Pazopanib	Alive at EOS, Day 572
18 -<50, ECOG PS 0	Doxorubicin/ifosfamide	30 mcg On treatment 2.2 months	PD at Day 61	None reported	Alive at EOS, Day 559
18 -<50, ECOG PS 1	Doxorubicin/ifosfamide	30 mcg On treatment 2.3 months	PD at Day 64	Pazopanib MAGE-A4 T cells	Alive at EOS, Day 544
18 -<50, ECOG PS 0	Doxorubicin/ifosfamide, doxorubicin, NY-ESO-1 T cells	100 mcg On treatment 2.1 months	PD at Day 59	Pazopanib MAGE-A4 T cells	Alive at EOS, Day 499
≥65, ECOG PS 1	Ifosfamide	30/100 mcg On treatment 3.8 months	BOR SD, PD at Day 117	Clinical trial (not otherwise specified)	Alive at EOS, Day 457
18 -<50, ECOG PS 1	Ifosfamide	30 mcg On treatment 12.4 months	BOR SD, PD at Day 238 with tumor shrinkage (-13%)	Ifosfamide/ etoposide	Death due to PD, Day 417
18 -<50, ECOG PS 0	Doxorubicin/ifosfamide, ifosfamide	30/100 mcg On treatment 7.4 months	BOR SD, PD at Day 113	None reported	Alive at EOS, Day 394
18 -<50, ECOG PS 0	Doxorubicin/ifosfamide	30/100/180 mcg On treatment 1.8 months	PD at Day 64	Pazopanib, trabectidin	Death due to PD, Day 355
18 -<50, ECOG PS 0	Doxorubicin/ifosfamide, durvalumab/tremelimumab (2×), NY-ESO-1 T cells (2×), pazopanib	100 mcg On treatment 2.1 months	PD at Day 59	Dacarbazine	Death due to PD, Day 295
18 -<50, ECOG PS 0	Ifosfamide / epirubicin (2×), MAGE-A4 T cells (2×), pembrolizumab, pazopanib, doxorubicin / ifosfamide	30/100 mcg On treatment 1.4 months	PD at Day 41	Ifosfamide	Death due to PD, Day 240
≥65, ECOG PS 1	Ifosfamide, MAGE-A4 T cells	30/100/180 mcg On treatment 1.9 months	PD at Day 58	None reported	Death due to PD, Day 144
18 -<50, ECOG PS 1	Doxorubicin/ifosfamide (2×), trabectidin	30 mcg On treatment 2.6 months	PD at Day 58	None reported	Death due to PD, Day 113

50 - <65, ECOG PS 0	Doxorubicin / ifosfamide, ifosfamide, trabectedin, MAGE-A4 T cells	30/100/180 mcg<br>On treatment 1.8 months	PD at Day 36	None reported	Alive at End of Study, Day 85
18 -<50	Doxorubicin/ifosfamide, pazopanib, NY-ESO-1 T cells	30/100/300 mcg planned; received 30/100. On treatment 0.5 months	Not assessed	None reported	Withdrew consent, Day 42

ECOG PS = Eastern Cooperative Oncology Group Performance Status; EOS = End of Study; NTL = non-target lesion; PD = progressive disease; SD = stable disease.
